# A Topological Characterization of Medium-Dependent Essential Metabolic Reactions

**DOI:** 10.3390/metabo2030632

**Published:** 2012-09-24

**Authors:** Nikolaus Sonnenschein, Carsten Marr, Marc-Thorsten Hütt

**Affiliations:** 1 School of Engineering and Science, Jacobs University Bremen, Campus Ring 1, 28759 Bremen, Germany; 2 Department of Bioengineering, University of California, San Diego, 9500 Gilman Drive, La Jolla, CA 92093, USA; Email: m.huett@jacobs-university.de; 3 Institute of Bioinformatics and Systems Biology, Helmholtz Zentrum München, German Research Center for Environmental Health, 85764 Neuherberg, Germany; Email: carsten.marr@helmholtz-muenchen.de

**Keywords:** flux-balance analysis, metabolic networks, network motifs

## Abstract

Metabolism has frequently been analyzed from a network perspective. A major question is how network properties correlate with biological features like growth rates, flux patterns and enzyme essentiality. Using methods from graph theory as well as established topological categories of metabolic systems, we analyze the essentiality of metabolic reactions depending on the growth medium and identify the topological footprint of these reactions. We find that the typical topological context of a medium-dependent essential reaction is systematically different from that of a globally essential reaction. In particular, we observe systematic differences in the distribution of medium-dependent essential reactions across three-node subgraphs (the *network motif signature* of medium-dependent essential reactions) compared to globally essential or globally redundant reactions. In this way, we provide evidence that the analysis of metabolic systems on the few-node subgraph scale is meaningful for explaining dynamic patterns. This topological characterization of medium-dependent essentiality provides a better understanding of the interplay between reaction deletions and environmental conditions.

## 1. Introduction

How topology shapes dynamics is a long-standing question in the field of network theory [1,2]. Many attempts have been formulated to understand the functional structure of metabolic networks from first principles using evolutionary, biochemical, or graph theoretical arguments [3,4,5,6,7,8]. Several works have argued that the network topology of metabolic systems is markedly optimized for robustness. For example, Marr *et al.* [9] used binary dynamic probes to demonstrate that on average fluctuations are dampened out in real metabolic networks. Also, there seems to be a selection for minimal metabolic pathways, given the experimental conditions [10]. The accessible nutrients for a species may thus be inferred by analyzing the network topologies. 

Furthermore, robustness of metabolism against gene or reaction deletions has been explored using flux-balance analysis (FBA) [11]. Particularly, its capacity to predict gene essentiality with high accuracy for *E. coli* and *Saccharomyces cerevisiae* has turned FBA into a widely accepted method for *in silico* studies of metabolic states [12,13]. More recent refinements of FBA focus on the redistribution of fluxes due to gene deletions [14,15].

Along similar lines of research, metabolic reactions have been classified in several ways based on topological information [3,16,17,18]. Here we will focus on two recent examples providing such classifications: UPUC (uniquely producing/consuming) and SA (synthetic accessibility) reactions.

UPUC metabolites have been introduced by Samal *et al.* [19]. They were described as metabolites that are consumed and produced by only a single reaction and, thus, exhibit the lowest possible degree in a bipartite network representation of the metabolic system (metabolites and reactions are both represented as inter- but not intra-connected node sets). A UPUC cluster may then be defined as a reaction subset that connects a set of UPUC metabolites. Besides the high essentiality of these UPUC reactions, which is one of the key issues in [19], they comprise also some other quite interesting features, e.g., proportionally fixed steady-state fluxes and significant correspondence with gene-regulatory modules [19]. We would like to point out that the UPUC category, as defined above, has not been used in the original study of Samal *et al.* [19], but rather a set consisting of reactions that are either associated with UP or UC metabolites.

Synthetic accessibility (SA), defined by Wunderlich and Mirny [20], is influenced by a measure used in chemical drug design describing the number of steps needed to synthesize a specific compound from a given set of compounds. Accordingly, the SA for a metabolic system is defined as the minimal number of reactions needed to reach a set of outputs (e.g., biomass) from a given set of inputs (e.g., medium composition) as obtained by a breadth-first-search traversal that can only proceed if all needed substrates are available. SA is successful in predicting essential genes, as many lethal mutations lead to an increase of the SA [20]. For this work we choose to treat SA as a reaction category by assigning an SA label to every reaction whose knock-out causes a change in biomass SA.

**Figure 1:**
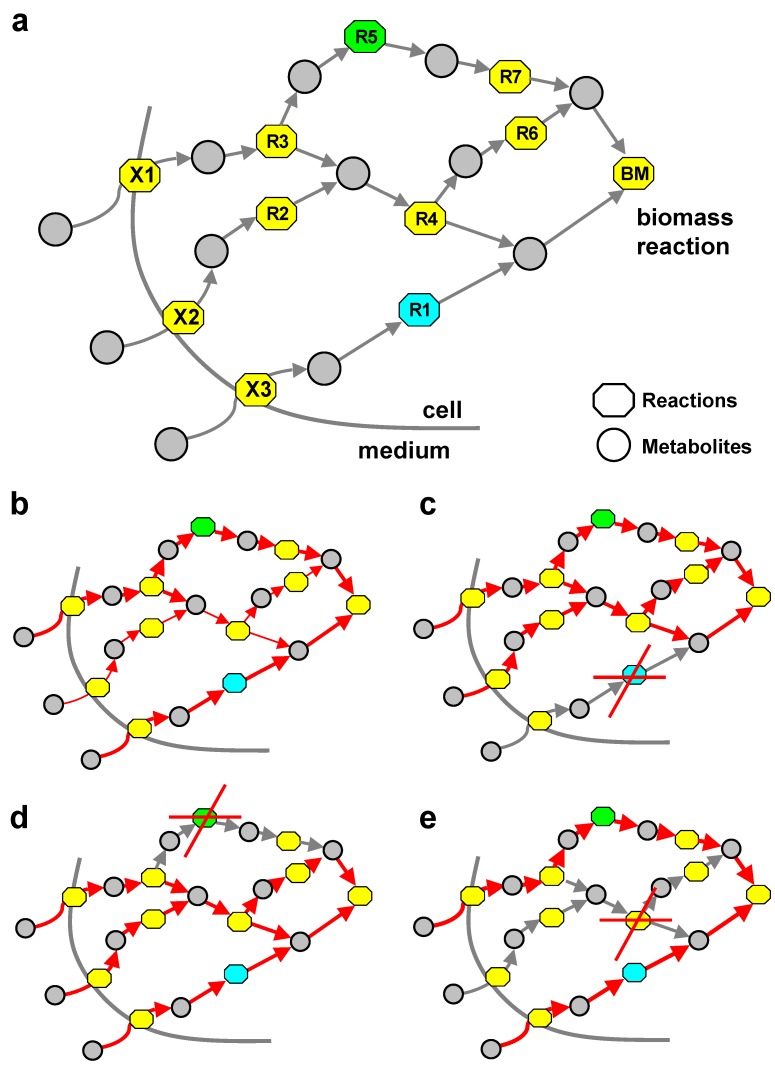
Network context of topological reaction categories. (**a**) Simple scheme of a small fictitious metabolic reaction system with examples of UPUC and SA reactions. (**b**) Wild-type network. (**c**) Knockout of SA reaction *R1*. Fluxes are rerouted over *R4* leading to an increase in the systems SA. (**d**) Knockout of UPUC reaction *R5*. (**e**) Knockout of reaction *R4*. *R1* (SA) and *R5* (UPUC) are now correct essentiality predictors. Edge thickness indicates flux magnitude.

Figure 1a shows a schematic representation of metabolism with three exchange reactions (*X*1, *X*2 and *X*3) with the environment and a two-component biomass reaction (*BM*). Circles represent metabolites, while boxes stand for reactions in this bipartite graph view of a metabolic system. In this Figure, *R*1 (highlighted in blue) is an example of an SA reaction, as it represents one of the shortest paths to *BM*, while *R*5 (highlighted in green) is consuming and producing only metabolites, which are uniquely produced (UP) and uniquely consumed (UC), and thus is an example of a UPUC reaction. Figure 1b–e provides a qualitative impression of the wild-type flux distribution (Figure 1b) and the re-routing of fluxes upon *R*1 and *R*5 knockout (Figure 1c,e), respectively.

In the example in Figure 1, both reactions (*R*1 and *R*5) have an alternative path that goes along reaction *R*4. Thus, both reaction labels would in this case not serve as a reliable predictor of the reaction’s essentiality. Eliminating reaction *R*4 (see Figure 1e) from the system would remove this alternative path, thus turning *R*1 and *R*5 into essential reactions. This (suggestive) example illustrates why a systematic study of combinatorial subsets of these categories can be interesting for understanding the topological basis of essential reactions.

A third category of reactions comes from a sampling of random environmental conditions and predicting steady-state fluxes that optimize biomass production using FBA. The set of reactions predicted to be active in all conditions has been termed metabolic core (MC) [21]. Remarkably, the MC and the other two topological reaction categories are all fairly accurate predictors of reaction essentiality.

Although experimental data from systematic knockout studies is available for *E. coli* [22,23], these essentiality profiles result from a limited set of environmental conditions. In particular, it has been pointed out recently that essentiality is often medium-dependent [24,25]. While this has been analyzed in [25] for genetic interactions (*i.e.*, the effect of a knockout under the condition of another knockout), we analyze here the above categories (SA, UPUC and MC reactions) in light of single-knockout mediumdependent essentiality.

An alternative approach of exploring the relationship between network architecture and function is based on the enumeration of few-node subgraphs. It has been shown that the subgraph composition of functionally related networks tends to be similar [26]. Also, in some cases, dynamical functions can be explained by small few-node subgraphs serving as devices for specific tasks organized locally in the graph. A potential signature of the functional role of few-node subgraphs is their statistical over- or under-representation (compared to a suitable ensemble of random graphs). Such subgraphs are called *network motifs*. This general concept has been introduced and developed by the Alon group [27,28], particularly for transcriptional regulatory networks [26,29], but not for metabolic networks. For an analysis of a network motif in the context of metabolism see [30]

Here we explore the question if a topological understanding of reaction essentiality can be established by integrating the *in silico* determined knock-out data with the three reaction categories and all combinatorial three-node subgraphs.

We start by introducing the *relative essentiality* of a reaction defined on the basis of a large number of combinatorial minimal media simulations. For each medium, the essentiality of all active reactions is tested *in silico*. In Section 2.1 the relative essentialities will be used as a basis of the three essentiality classes: always essential (*essential*), essential only in some growth media (*conditional lethal*), and never essential (*non-essential*).

Section 2.2 is devoted to an initial analysis of the three categories of reactions (UPUC, SA and MC). We present an exhaustive analysis of all combinatorial sets of reactions formed out of these three categories, arriving at a refined topological characterization of medium-dependent essential reactions.

Section 2.3 is devoted to exploring the distribution of essentiality classes and established topological categories across three-node subgraphs of the reaction-centric metabolic network.

In Section 4 we interpret the findings from Section 2.2 and Section 2.3 and use them to topologically characterize a typical reaction displaying medium-dependent essentiality.

See Table 1 for a summary of model, reaction category, activity, and essentiality statistics.

**Table 1:** Summarizing statistics of reaction categories and essentialities.

	Reactions	UPUC	SA	MC	No category
Overall/Cytosol	1284/707	296/193	238/230	231/197	820/375
Non-essential (Overall/Cytosol)	789/402	137/57	27/25	25/16	608/312
Conditional lethal (Overall/Cytosol)	326/162	90/74	73/73	85/79	198/55
Essential (Overall/Cytosol)	169/143	69/62	138/132	121/102	14/8

## 2. Results

### 2.1. Relative Essentiality Analysis

In order to subdivide the metabolic reactions into essentiality classes, namely *non-essential*, *conditional lethal*, and *essential*, we quantify the *relative essentiality* of a reaction by computing optimal, *i.e.*, maximizing biomass production, steady-state flux distributions for over more than 7 × 10^4^ combinatorial minimal media conditions. Furthermore, all subsequent single reaction knockouts of active (non-zero flux carrying) reactions are performed to identify for each medium condition the set of essential reactions (see Methods for details). An illustrative example of this concept, involving *E. coli* central carbon metabolism [31], is provided in the supplementary materials.

The relative essentiality of a particular reaction is then defined as the number of lethal outcomes due to its removal divided by the number of environmental conditions under which it has been active. An alternative definition of relative essentiality would be to normalize the number of lethal outcomes to the total number of media sampled. In this case, however, essential reactions that are rarely active would give an unrealistically low essentiality value.

**Figure 2:**
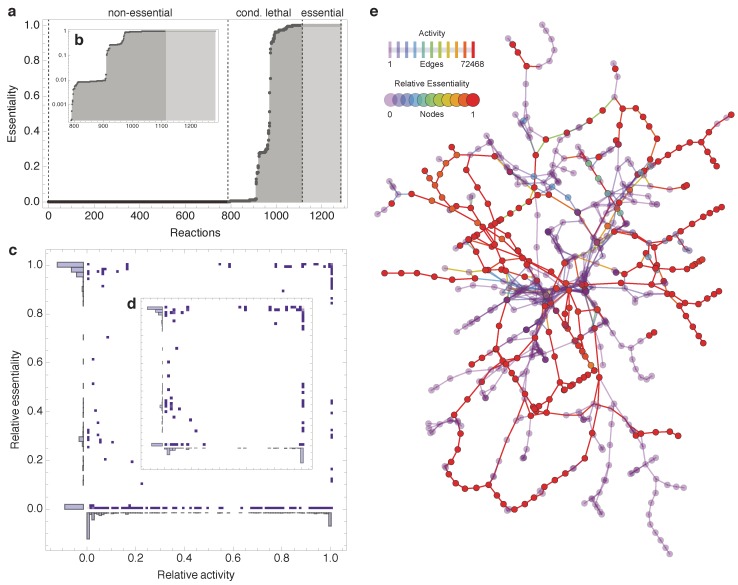
Outcome of combinatorial minimal media simulations. (**a**) Sorted relative essentiality profile determined by the simulation of reaction deletions under 72468 combinatorial minimal media conditions. The three different essentiality classes are indicated by dashed lines. Inset (**b**) shows the same profile using semilog plot. (**c**) No relation is observed between essentiality and activity (*non-essential* reactions have been removed in inset (**d**); relative activity is the number of simulations that a reaction was active normalized by the total number of simulations). (**e**) A reaction-centric network diagram illustrating the relative essentiality on the nodes (707 reactions) and co-occurring activity on the edges of the cytoplasmic part of the *i*AF1260 model (transport, periplasmic, and blocked reactions have been discarded; currency metabolite have been removed manually, see Methods).

Figure 2a shows the sorted relative essentiality profile of all reactions in the *E. coli* model, which have been active at least once during the FBA simulations; blocked reactions [32] have thus been eliminated; see also Methods). In Figure 2a the three essentiality classes are clearly visible: The removal of most reactions has no or only small consequences for the production of biomass (*non-essential*). Some reactions are globally essential (*essential*) and a third set is only medium-dependent essential (*conditional lethal*). Excluding *non-essential* reactions, Figure 2b depicts the relative essentiality profile in a semilog plot. The stepwise appearance of the *conditional lethal* curve indicates three major groups of reactions that exhibit very similar relative essentiality scores, connected by some intermediate reactions.

Almaas *et al.* [21] reported a high, global essentiality for MC reactions. This finding suggests a higher essentiality for more active reactions. However, the activity and relative essentiality profiles exhibit no such correlation (see Figure 2c,d). Moreover, in contrast to [21], MC reactions are not exclusively *essential*. This deviation from previously reported results seems to be due to model differences (Almaas *et al.* utilized the older *E. coli* model *i*JR904 [33]; see Supplementary Figure S2) and not due to the changes in simulation procedures (combinatorial minimal media in our study *vs.* random media sampling in [21]; see Supplementary Figure S3).

Figure 2e displays the computed relative essentiality profile as well as the co-occurrence frequency of all cytosolic reactions in *E. coli* metabolism. Albeit visually appealing, no clear pattern emerges from this type of visualization, substantiating the need for a more rigorous topological analysis.

### 2.2. Topological Categories as Markers of Essential Reactions

**Figure 3:**
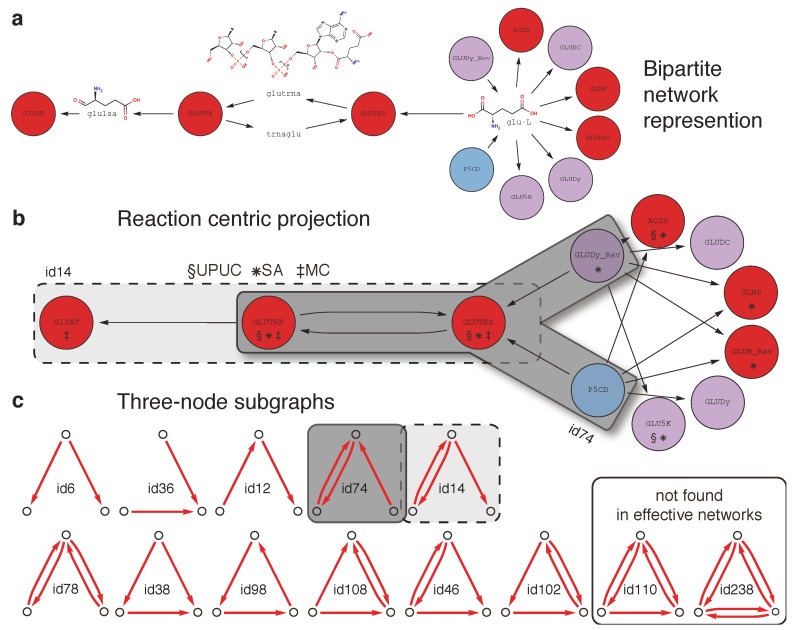
(**a**) Bipartite network representation of the two step conversion of L-glutamate (*glu-l*) into L-glutamate-1-semialdehyde (*glu1sa*) involving glutamyl-tRNAsynthetase (*GLUTRS*; 6.1.1.17) and glutamyl-tRNA reductase (*GLUTRR*; 1.2.1.-). Additionally, two reaction sources for L-glutamate, Δ^1^-pyrroline-5-carboxylate dehydrogenase (*P5CD*; 1.5.1.12) and the reverse direction of glutamate dehydrogenase (*GLUDy_Rev*; 1.4.1.4), and one sink for L-glutamate-1-semialdehyde, glutamate-1-semialdehyde aminotransferase (*G1SAT; 5.4.3.8*), are shown, among other reactions consuming L-glutamate. Node colors indicate relative essentiality (legend provided in Figure 2). (**b**) A reaction centric projection of the bipartite network in (**a**). Reaction categories (UPUC, SA, and MC) are shown for each reaction node and gray boxes indicate the occurrence of one subgraph of type *id14* and two subgraphs of type *id74* (see **c**). (**c**) All (13) combinatorial three-node subgraphs and corresponding identifiers (*id110* and *id238* have not been encountered in any effective network).

Figure 3 illustrates the rationale behind our investigation by summarizing the diverse topological representations of groups of metabolic reactions. These different representations (bipartite network representation → reaction-centric representation → established topological categories and three node subgraphs) are compared, either individually or in combination, with the essentiality classes introduced above, in order to understand typical topological “implementations” of *conditional lethal* reactions.

**Figure 4:**
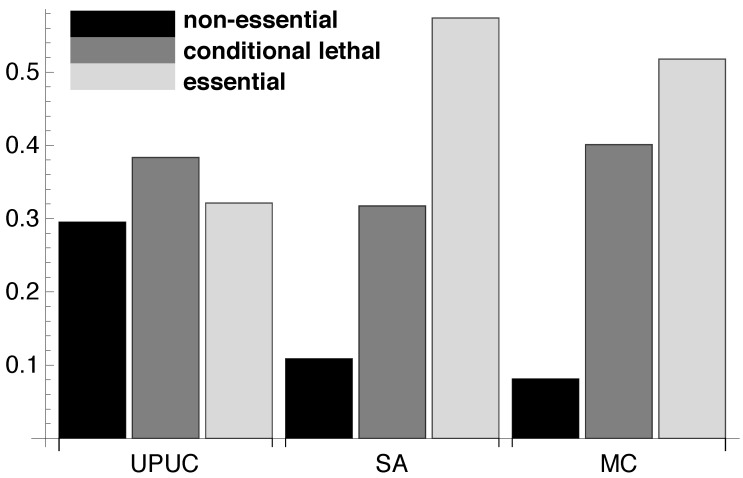
Reaction categories and essentiality classes. The proportions of the three different essentiality classes determined for UPUC, SA and MC component (for absolute numbers see Supplementary Figure S7).

Using the relative essentiality profile (see Figure 2), we determine the amount of reactions belonging to the three essentiality classes for each of the three reaction categories. The results in Figure 4 show that the three categories incorporate different amounts of reactions belonging to each of the three essentiality classes. The SA reaction set seems to be composed of a mixture of *conditional lethal* and *essential* reactions whereas the UPUC reactions exhibit a high amount of *non-essential* reactions. As already mentioned, in contrast to previous findings ([21], see previous Section 2.1), the MC is not exclusively composed of *essential* reactions, but rather shows a similar essentiality class composition as the SA category.

**Figure 5:**
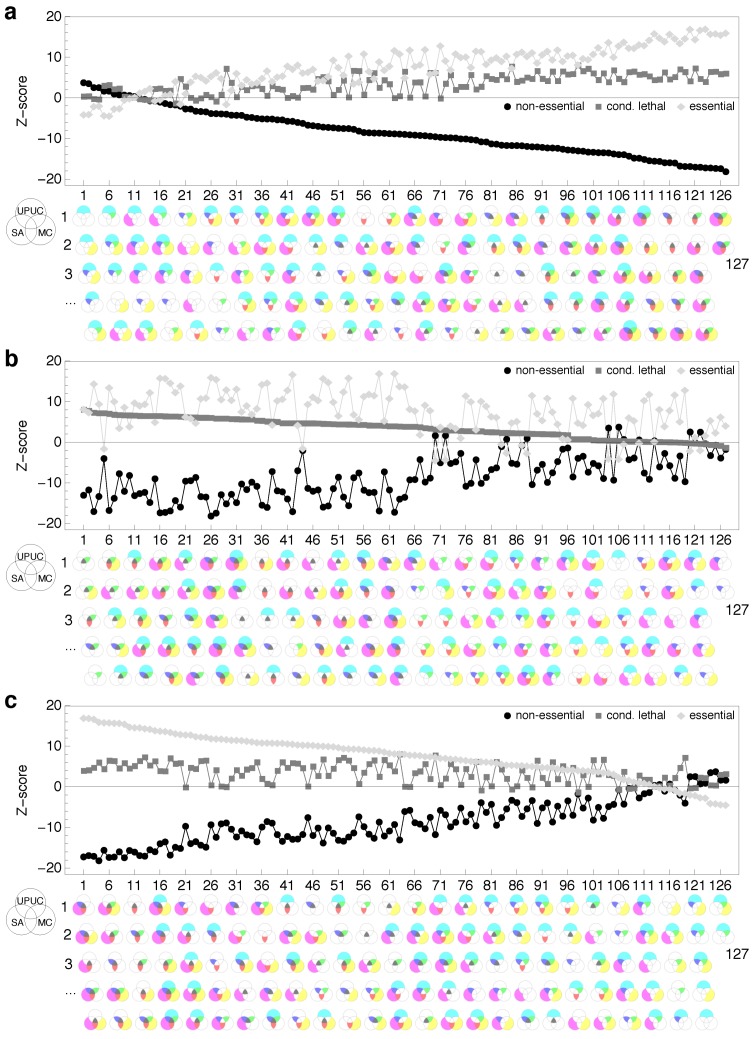
Enrichment of combinatorial reaction category sets for essentiality classes. Combinatorial sets sorted on the basis of (**a**) *non-essential*, (**b**) *conditional lethal* and (**c**) *essential* class enrichment. Venn diagrams [34] on the abscissa indicate each of the 127 nonempty unions and intersections of UPUC (upper circle), SA (lower left circle) and MC (lower right circle), respectively (no set includes less than 13 reactions, the largest combinatorial set includes 332 reactions). For the computation of Z-scores, random distribution of essentiality classes among the Venn intersections and unions was used as the underlying null hypothesis.

Next, we explore the composition of essentiality classes in combinatorial sets of reaction categories (see Figure 5). For all 127 non-empty intersections and unions of UPUC, SA and MC, we perform an enrichment analysis for the three reaction essentiality classes. Besides a few exceptional cases, *non-essential* reactions seem to be strongly underrepresented in the majority of combinatorial sets (Figure 5a), coinciding with the capability of these reaction categories to predict essentiality. The exceptions to this observation have a tendency to include the exclusively UPUC set *UPUC*∩*SA^c^*∩*MC^c^* where *X^c^* denotes the absolute complement. On the other hand, the majority of combinatorial sets is significantly enriched for *essential* reactions (Figure 5c). More importantly, more than half of the combinatorial sets exhibit a clear separation of *essential* from *conditional lethal* and *non-essential* classes. Comparing Figure 5a and Figure 5c reveals that the sequence of combinatorial sets in the sorted *non-essential* enrichment resembles the *essential* sequence in reverse order (e.g., the exclusive UPUC set being visually absent for high *essential* reaction enrichment). This observation provides evidence for a strong negative association between these two essentiality classes in the context of the UPUC, SA and MC categories.

Unfortunately, no clear separation of *conditional lethal* from *non-essential* and *essential* reactions is achieved by this combinatorial approach (Figure 5b). These results indicate that UPUC, SA and MC, albeit good essentiality predictors, do not provide the means for a topological characterization of medium-dependent essentiality.

### 2.3. Distribution of Essentiality Classes Across Three-Node Subgraphs

In the following we will now quantify whether the established topological categories or the three-node subgraphs contain more information about medium-dependent essential reactions.

**Figure 6:**
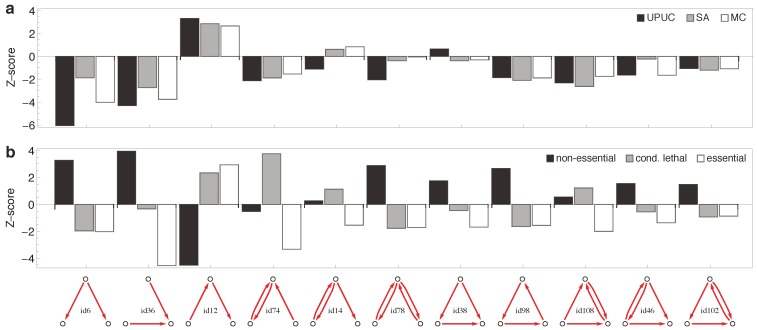
Enrichment on three-node subgraphs. The statistical over- and under-representation of (**a**) reaction categories and (**b**) essentiality classes on all occurring three-node subgraphs (two motifs have been omitted as they were not detected in any effective network). For the computation of Z-scores, random distribution of reaction categories and essentiality classes among subgraphs was used as underlying null hypothesis.

Figure 6 shows the statistical over- and under-representation of the three established topological categories (Figure 6a) and the three essentiality classes (Figure 6b) across all possible three-node subgraphs of the reaction-centric metabolic network (Figure 3). The striking result is that the three established topological categories display very similar subgraph associations, while the three essentiality classes show strong differences in their subgraph associations. Counter-intuitively, subgraphs thus perform better in distinguishing essentiality classes than in distinguishing the established topological categories discussed above.

*Conditional lethal* reactions have a fundamentally different “footprint” when mapped onto subgraphs. The most important building block is the bidirectional V-in (*i.e.*, the V-in with one of the links being bi-directional). *Non-essential* reactions, on the other hand, are suppressed in chains, but elevated in V-in and V-out subgraphs. Surprisingly, all the topology-motivated reaction categories (UPUC, SA, MC) display very similar occurrence patterns across the motifs.

In contrast to the majority of works on network motifs, we do not take the motif composition of the total (“static”) network into account, but rather compute the subgraph associations medium by medium from each *effective* network spanned by all reactions with non-zero flux optimizing biomass production. Supplementary Figure S4 shows the same analysis as Figure 6, but for the subgraphs extracted from the total, static network. It is seen that the signal (e.g., the discrimination between essentiality classes) is much weaker there.

This is conceptually more plausible since the reactions comprising a subgraph in the static network may in fact be never active together and, consequently, such a subgraph may functionally never be available (see Supplementary Figure S5 for a distribution of Hamming distances between subgraph occurrence profiles from the static and effective networks).

The topological “footprint” of the different essentiality classes cannot be affected by the number of occurrences of three-node subgraphs in the metabolic network, as the null model of randomly drawn sets of reactions compensates for this. It could be, however, that the clustering of reactions in one of the reaction categories or a bias in the degree distribution may induce a systematic skew in the distribution of these reactions over the three-node subgraphs. We checked for these distortions of our result by computing the amount of clustering in each of the essentiality classes (see Supplementary Figure S6).

The clustering is defined by the conditional probability of a reaction *r* being in this class *C* (e.g., conditional lethal) given that a neighboring reaction *r'* is in this class: *c*(*C*) = *P*(*r* ∈ *C*|*r'* ∈ *C*) = *P*(*r*, *r'* ∈ *C*)/*P*(*r'* ∈ *C*), *r'* ∈ *N*(*r*). *Essential* reactions exhibit the highest amount of clustering, but *non-essential* and *conditional lethal* reactions show very similar distributions (see Supplementary Figure S6). On this basis we expect that the results shown in Figure 6 are not severely distorted by clustering.

## 3. Methods

### 3.1. Metabolic Model and Network Representations

The genome-scale metabolic reconstruction *i*AF1260 [37] of *E. coli* was used in all our experiments. Each reversible reaction was replaced by two irreversible reactions acting in opposite directions. For our topological analyses, first a bipartite graph representation was generated from the stoichiometry of the model and then projected onto a reaction centric network (see [38] for a review on network representations of metabolism).

### 3.2. Flux-Balance Analysis

For a given metabolic model, flux-balance analysis (FBA) [11] enables the computation of a steady-state flux distribution that maximizes a specific biological objective *Z* (e.g., maximal biomass production). Generally, the linear optimization problem defined in FBA can be stated as follows:


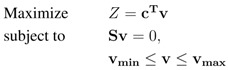
(1)

with an objective coefficient vector **c**, the stoichiometric matrix **S**, the flux vector **v** and the constraint vectors **v_min_** and **v_max_**. As we are considering reversible reactions as two independent unidirectional reactions, we set **v_min_** to zero. Problems like Equation 1 can be efficiently solved using linear programming. In order to avoid thermodynamically infeasible loops, we utilized pFBA [39], effectively using the solution of Equation 1, to fix the objective to its maximum value and minimize the L1-norm of all other fluxes in a second optimization.

### 3.3. Combinatorial Minimal Media and Reaction Essentiality

Combinatorial minimal media were constructed using the following procedure. (i) All experimentally verified nutrients in the *i*AF1260 model were classified as sources for elemental carbon, nitrogen, sulfur and phosphate (see also Supplementary Table S1). Some compounds fall hereby into multiple categories, e.g., glucose-6-phosphate is both a carbon and a phosphate source. (ii) Combinations of nutrients were then chosen such that only one of each elemental source was included in the medium, e.g., no additional phosphate source was provided in a medium containing glucose-6-phosphate. Steady-state fluxes that optimize biomass production have been calculated for all possible substrate combinations leading to a total of 72468 analyzed minimal-media conditions.

For each simulation, the essentiality of all active reactions was determined by fixing the respective fluxes to zero and recomputing the maximal biomass flux for the mutants. A reaction was classified as essential if the biomass flux dropped to zero.

### 3.4. Blocked Reactions

We removed all globally blocked reactions from the model to give the topological methods described in this article (UPUC, SA) the opportunity to work on the same information content as their dynamical counterpart (MC). A high (not as high as the default flux boundaries *v_max_*) maximal uptake and secretion rate was assigned to all available transporters in the system and then blocked reactions were confirmed by flux variability analysis [32]. These globally blocked reactions cannot carry a flux under any environmental conditions and consequently are not available to methods that use FBA.

### 3.5. Metabolic Core

Reactions are assigned to the metabolic core if they were active in all wild-type simulation, following the definition of Almaas *et al.* [21]. In contrast to [21], however, we use a finite number of combinatorial minimal media instead of randomly sampled conditions. Consequently, the size of the metabolic core in this study is larger than in the original work.

### 3.6. Synthetic Accessibility Reactions

The synthetic accessibility of all reactions in the system was computed according to [20]. The needed outputs were defined to be the substrates of the biomass function and the ingredients of a glucose minimal medium were defined to be the inputs of the system. As a variation to [20] we decided to include no further additional compounds that ensure that all outputs are reached in the wild type. Instead we used a set of bootstrapping metabolites [40] that permit a proper functioning of the algorithm but are not the starting points of the breadth first search.

### 3.7. UPUC Reactions

The UPUC reactions were determined in analogy to the algorithm published in [19]. We determined all metabolites with an in-degree and out-degree of one (UPUC metabolites) in the bipartite graph representation of the metabolism of *i*AF1260. Then we computed the set of reactions (UPUC reactions) that are associated with the set of UPUC metabolites for further analysis.

### 3.8. Enumeration of Three-Node Subgraphs

Three-node motifs as well as static networks were enumerated using the software *mfinder* [28]. There are two sorting schemes for subgraph types in the literature. We employed the one from Milo *et al.*, where subgraphs are grouped according to criteria (cyclic *versus* acyclic; then connectivity or number of bidirectional links), rather than the one, where three-node subgraphs are sorted according to their “identifier” (the adjacency matrix of the subgraph, read as a binary number). In all subgraph-related figures, this subgraph identifier is also indicated in the corresponding subgraph pictogram.

## 4. Conclusions

Using a variety of topological descriptors, we have been able to characterize the network properties of reactions displaying medium-dependent essentiality in a large-scale combinatorial minimal media screen employing flux-balance analysis.

The two classification schemes for metabolic reactions are (1) occurrence in directed three-node subgraphs and (2) functional categories of metabolic reactions motivated by network topology and FBA. We observe that the distribution of the three classes of metabolic reactions derived from relative essentiality is non-random across the three-node subgraphs. At the same time the distribution of essentiality classes across the three functional categories (UPUC, SA and MC) is highly diverse for the conditional lethal reactions, but far more homogeneous for the other two classes. Putting all these observations together leads to an accurate topological characterization of medium-dependent essential reactions.

These two topological characterizations are quite independent. In particular, when distributing the reaction categories across the three-node subgraphs, we see almost no differences between the three reaction categories in their subgraph preference profile.

Among the diverse combinatorial sets defined from the established topological categories, several very different ones contain a large number of *conditional lethal* reactions, suggesting different sub-categories of these medium-dependent essential reactions. We believe that this method of exploring combinatorial sets defined from multiple topological labels with the goal of investigating the relationship between network properties and system properties may be helpful in a broad range of contexts in systems biology.

With the wide range of FBA models available, a natural next step of our investigation is to analyze these categories of essential reactions and their topological implementation also in other organisms. Also, other categories of metabolic reactions derived from large FBA screens can be topologically assessed, for example reactions that are active only in a very small number of environmental conditions (rarely active reactions). We expect that the topological implementation of such rarely active reactions can shed light on the robustness of metabolic systems against environmental variations.

Lastly, further validating the results with gene expression data can be an interesting line of investigation, starting from our previous work on effective networks derived from gene expression patterns [35] and a network interpretation of reactions contributing to *metabolic inconsistency* (*i.e.*, to mismatches between gene expression data and predicted metabolic flux patterns; see [36]).

## Supplementary Files

Supplementary File 1 PDF-Document (PDF, 758 KB)
