# Supplementary material for: A Topological Characterization of Medium-Dependent Essential Metabolic Reactions
Source: Metabolites. 2012 Sep 24;2(3):632–47. doi: 10.3390/metabo2030632 (PMC3901215; doi:10.3390/metabo2030632)
Supplement: Supplementary File 1 — PDF-Document (PDF, 758 KB) [file metabolites-02-00632-s001.pdf]

1. Relative Essentiality—An Illustrative Example

As an illustrative example of medium-dependent essentiality (*i.e.*, relative essentiality arising from monitoring essentiality across a large set of media) we want briefly discuss the central metabolism model for *E. coli* (*E. coli* core model, see [31]) as a minimal system and study the predicted biomass production under knockout for seven different carbon sources (glucose, acetate, fumarate, lactose, succinate, ethanol, pyruvate). The FBA model consists of 63 reactions, including 14 exchange reactions, which also regulate the uptake of the respective carbon source provided. FBA studies for this system have been performed using the 15 component biomass function provided with the model [31]. Figure S1a shows the size of the biomass flux for different mutants (bars) for each medium (color segments in each bar). As expected, the biomass flux is typically largest for the glucose medium. In one case (*i.e.*, the removal of *GLCpts*, which is a glucose transport reaction), however, only other carbon sources lead to a non-zero biomass flux prediction. The relative sizes of the color segments (*i.e.*, of the biomass flux under a particular carbon source) vary greatly from mutant to mutant.

For example, the removal of the reaction FBP, which is catalyzed by the enzyme fructose-1,6-bisphosphatase and dephosphorylates fructose-1,6-bisphosphate to fructose-6-phosphate, is essential for all carbon sources except glucose. As a matter of fact, FBP is a step in gluconeogenesis, which is a pathway that generates glucose. In the case of the glucose medium, there is no need to generate glucose from other substances as it is provided directly via the glucose uptake reaction.

We have to mention that *in vivo* the deletion of the gene fbp is not lethal in *E. coli* as it can bypass the loss through other pathways. The deviant result comes about due to the restricted minimal model used in this illustrative example covering only central metabolism. As we proceed to the genome-scale reconstruction *i*AF1260 we find that FBP is actually never essential, in agreement with experimental results [22,23].

Figure S1. *E. coli* core model—medium dependent essentiality for seven carbon sources under aerobic conditions. The height of the bars indicates the size of the growth flux as determined by FBA. The reactions have been sorted according to the glucose medium biomass fluxes obtained from the reaction knockouts.

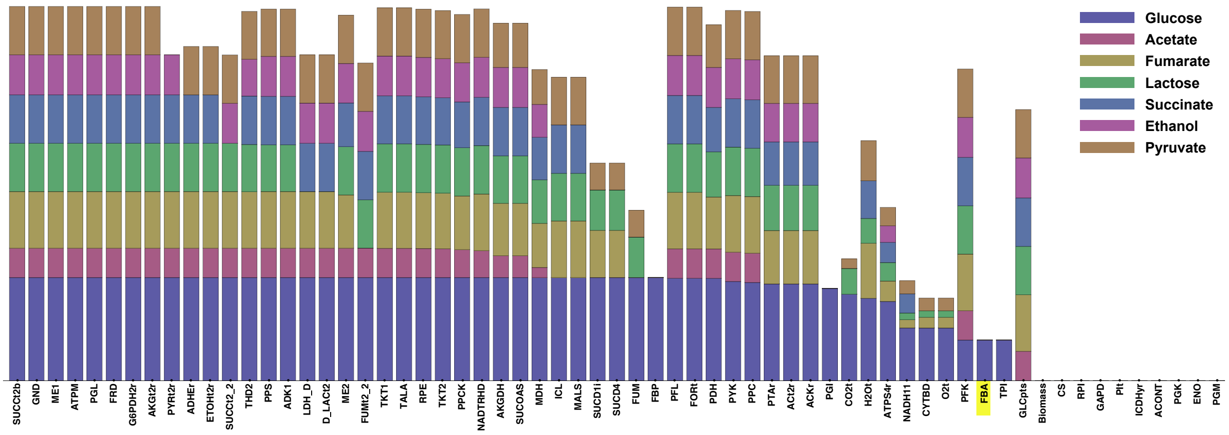

Figure S2. Reaction categories and essentiality classes for iJR904 and random media sampling. The proportions of the three different essentiality classes determined for UPUC, SA and MC component.

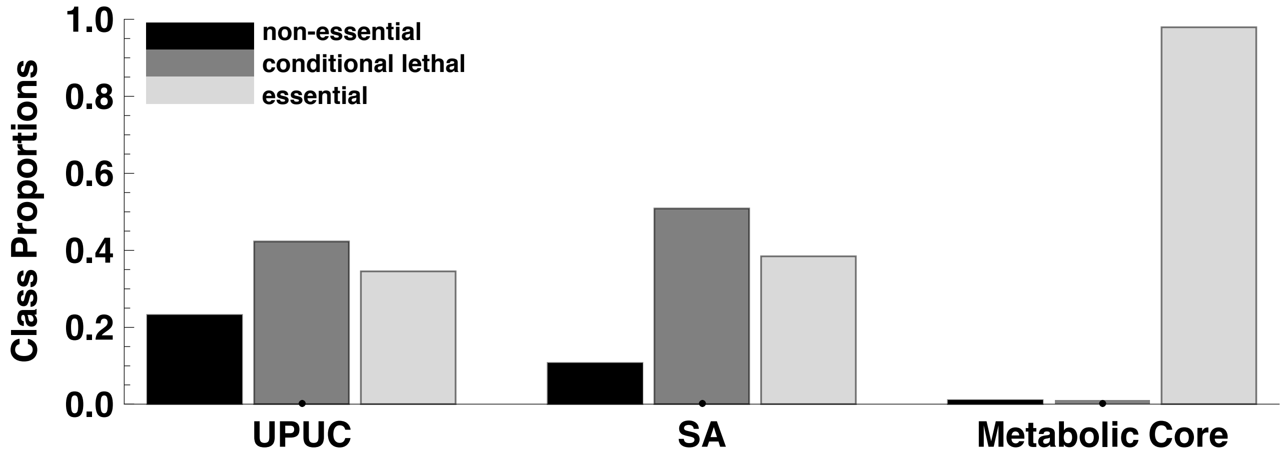

Figure S3. Reaction categories and essentiality classes for iAF1260 and random media sampling. The proportions of the three different essentiality classes determined for UPUC, SA and MC component.

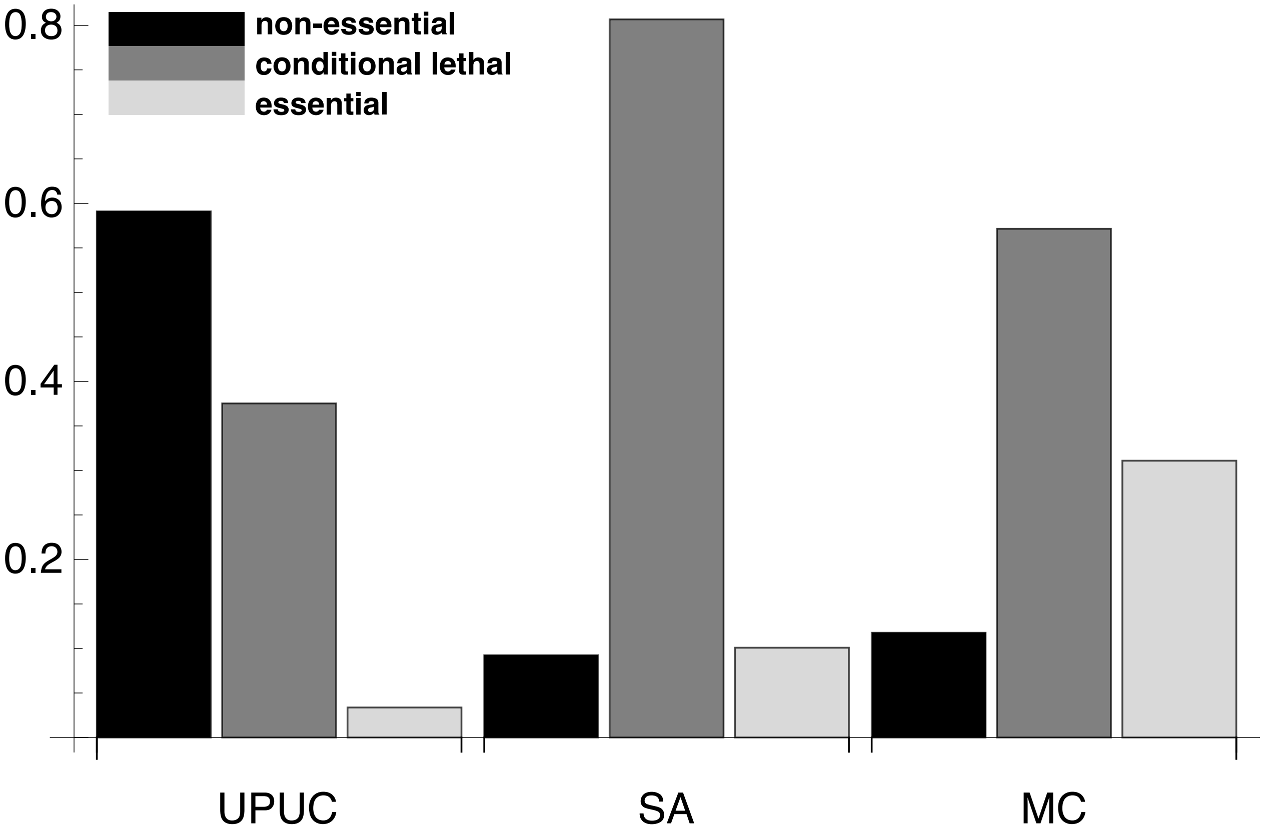

Figure S4. Category and class enrichment of three-node subgraphs. The distribution of (a) essentiality classes and (b) topological categories.

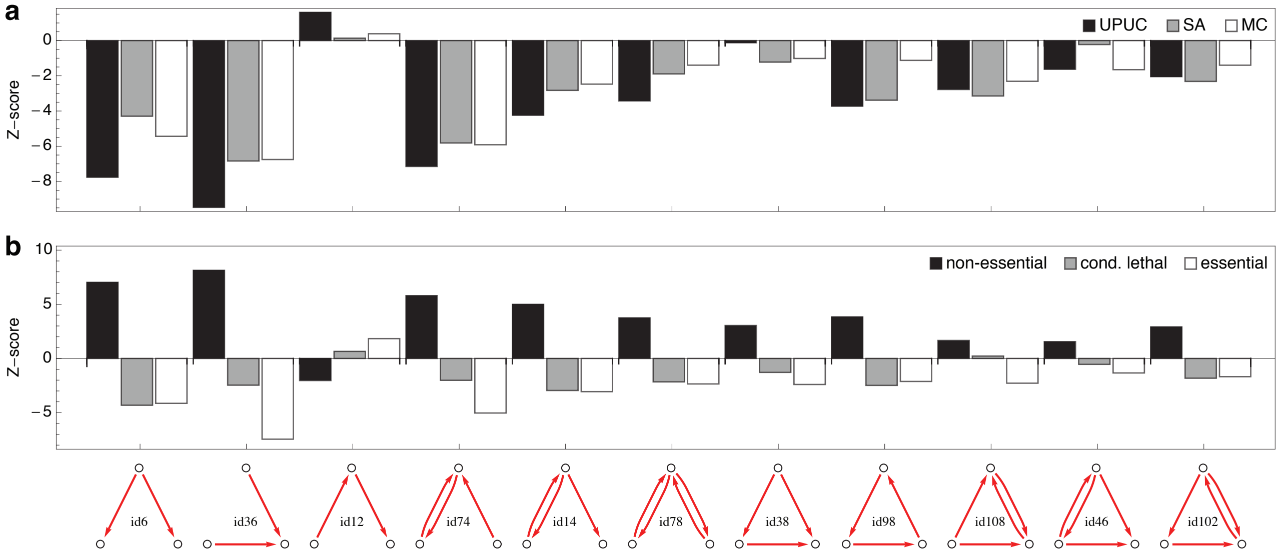

Figure S5. Distribution of Hamming distances between static and effective network subgraph profiles.

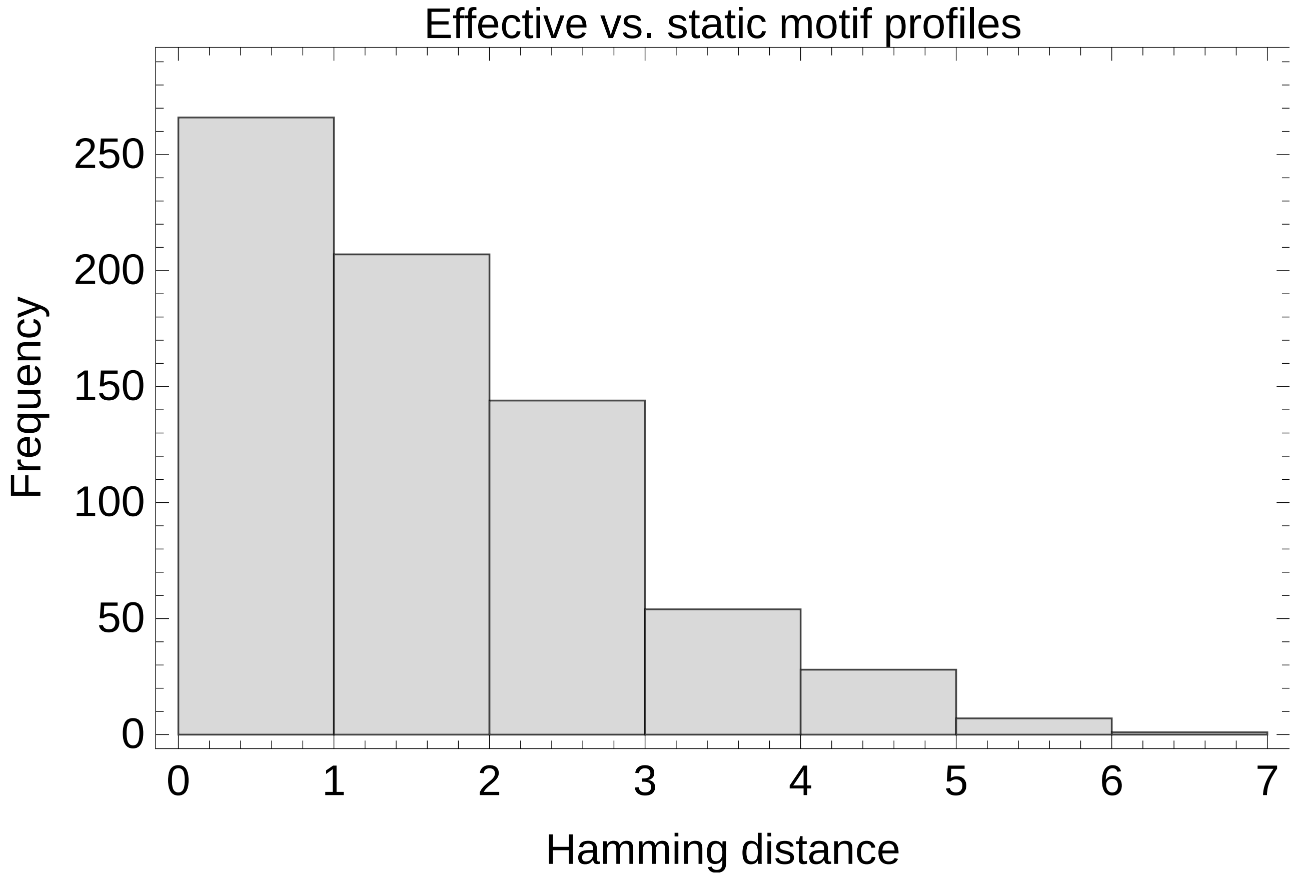

Figure S6. Distribution of clustering among the three essentiality classes.

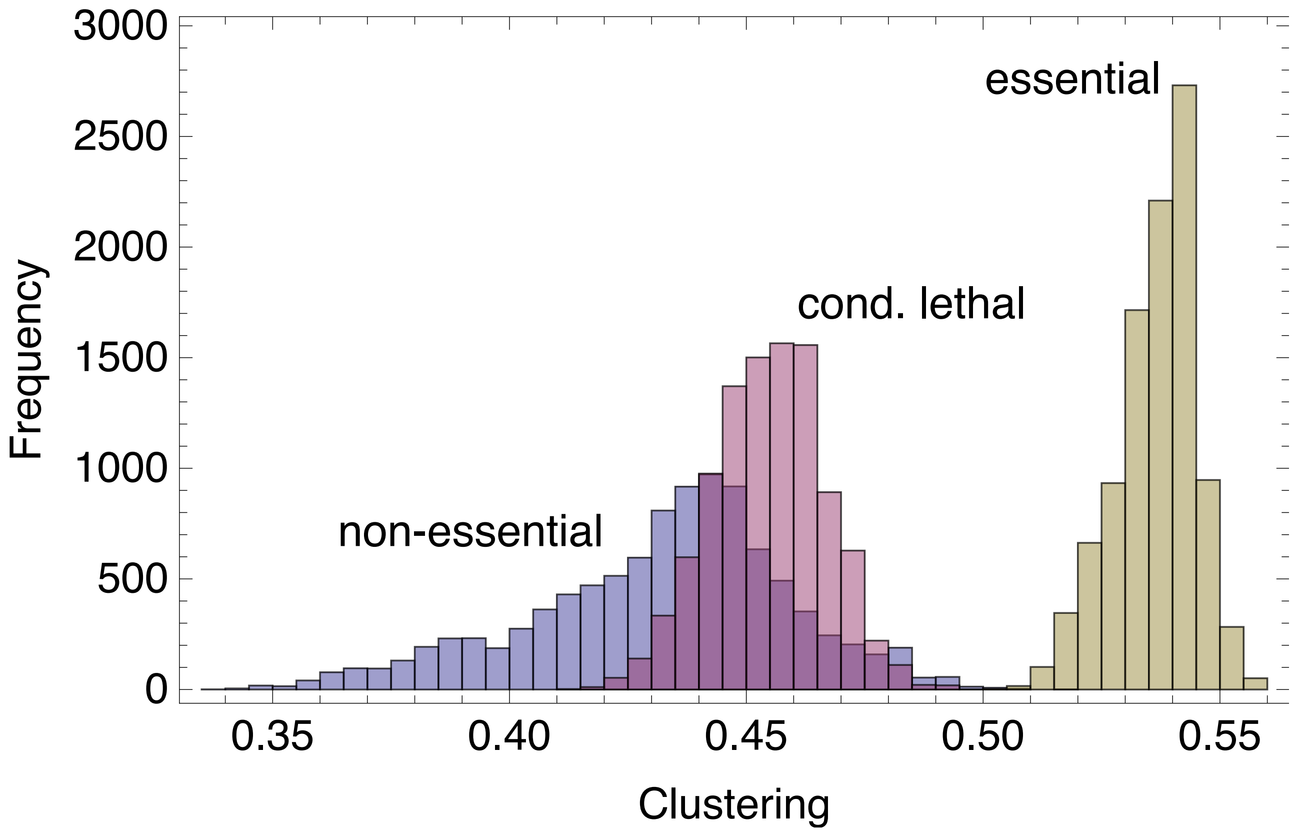

Figure S7. Reaction categories and essentiality classes. The absolute numbers of the three different essentiality classes determined for UPUC, SA and MC component (see also Figure 4 in the main manuscript).

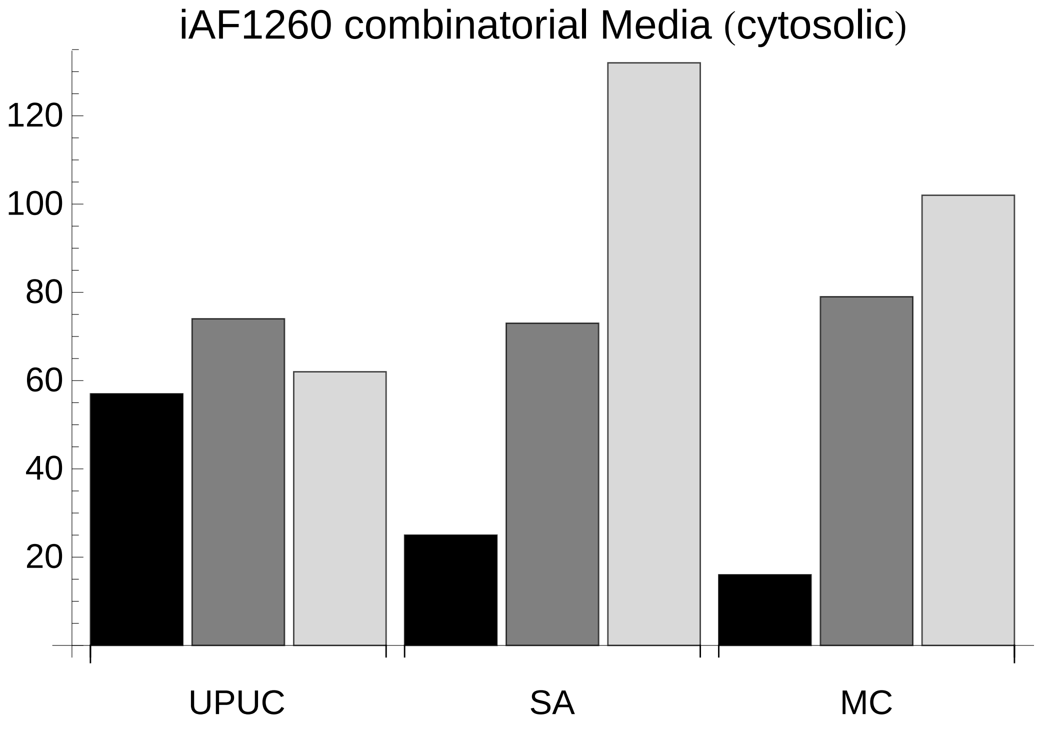

Table S1. Elemental composition of sources used for combinatorial minimal media generation. Only sources that have been validated by Biolog experiments have been used.

Source	Carbon	Nitrogen	Phosphor	Sulfur
ser-L	1	1	0	0
ser-D	1	1	0	0
asp-L	1	1	0	0
ala-D	1	1	0	0
acgam	1	1	0	0
g6p	1	0	1	0
g1p	1	0	1	0
xyl-D	1	0	0	0
uri	1	0	0	0
tre	1	0	0	0
thymd	1	0	0	0
succ	1	0	0	0
sbt-D	1	0	0	0
rmn	1	0	0	0
rib-D	1	0	0	0
pyr	1	0	0	0
mnl	1	0	0	0
melib	1	0	0	0
man	1	0	0	0
malttr	1	0	0	0
malt	1	0	0	0
mal-L	1	0	0	0
mal-D	1	0	0	0
lcts	1	0	0	0
lac-L	1	0	0	0
ins	1	0	0	0
glcur	1	0	0	0
glcr	1	0	0	0
glcn	1	0	0	0
glc-D	1	0	0	0
galur	1	0	0	0
galctn-L	1	0	0	0
galctn-D	1	0	0	0
galct-D	1	0	0	0
gal	1	0	0	0
fum	1	0	0	0
fucL	1	0	0	0
fru	1	0	0	0
f6p	1	0	0	0
dad2	1	0	0	0
arab-L	1	0	0	0
all-D	1	0	0	0
akg	1	0	0	0
adn	1	0	0	0
acnam	1	0	0	0
5dglcn	1	0	0	0

Table S1. *Cont.*

Source	Carbon	Nitrogen	Phosphor	Sulfur
cys-L	0	1	0	1
xtsn	0	1	0	0
xan	0	1	0	0
trp-L	0	1	0	0
thr-L	0	1	0	0
ptrc	0	1	0	0
pro-L	0	1	0	0
orn	0	1	0	0
nh4	0	1	0	0
gsn	0	1	0	0
gly	0	1	0	0
glu-L	0	1	0	0
gln-L	0	1	0	0
gam	0	1	0	0
cytd	0	1	0	0
csn	0	1	0	0
asn-L	0	1	0	0
arg-L	0	1	0	0
ala-L	0	1	0	0
acmana	0	1	0	0
ump	0	0	1	0
tyrp	0	0	1	0
thrp	0	0	1	0
pser-L	0	0	1	0
pi	0	0	1	0
man6p	0	0	1	0
gmp	0	0	1	0
gam6p	0	0	1	0
cmp	0	0	1	0
3ump	0	0	1	0
3gmp	0	0	1	0
3cmp	0	0	1	0
3amp	0	0	1	0
23cump	0	0	1	0
23cgmp	0	0	1	0
23ccmp	0	0	1	0
taur	0	0	0	1
so4	0	0	0	1
mso3	0	0	0	1
isetac	0	0	0	1
cys-D	0	0	0	1
butso3	0	0	0	1
